# Can a Good Break Shot Determine the Game Outcome in 9-Ball?

**DOI:** 10.3389/fpsyg.2021.691043

**Published:** 2021-07-29

**Authors:** Jing Wen Pan, John Komar, Shawn Bing Kai Sng, Pui Wah Kong

**Affiliations:** ^1^Physical Education and Sports Science Academic Group, National Institute of Education, Nanyang Technological University, Singapore, Singapore; ^2^Office of Graduate Studies and Professional Learning, National Institute of Education, Nanyang Technological University, Singapore, Singapore

**Keywords:** cue ball position, ball distribution, Voronoi diagram, pool, billiards

## Abstract

This study aimed to quantify the break shot characteristics and identify their significance in predicting the game outcomes in 9-ball tournaments. The break shots of 275 frames (241 men’s, 34 women’s) of professional tournaments were analyzed from two aspects: (1) cue ball position, represented by the distance between the cue ball and the table center, and (2) ball distribution, indicated by the standard deviation of Voronoi cell areas determined from all remaining balls on the table. Spearman correlation and binary logistic regression were utilized to identify associations and to predict the frame outcomes, respectively. Results showed that the more balls falling into the pockets during the break, the more clustered the remaining balls (*r*_*s*_ = 0.232, *p* < 0.001). The closer the cue ball ending toward the table center, the more balls potted in the visit immediately after the break (*r*_*s*_ = −0.144, *p* = 0.027). Neither cue ball position nor ball distribution could predict table clearance or winning of a frame. In conclusion, pocketing more balls during the break is associated with more clustered balls remaining on the table. Parking the cue ball near the table center after the break can facilitate potting more balls immediately after.

## Introduction

Nine-ball is a popular billiard game played with a cue stick on a rectangular table with six pockets. Players strike the white cue ball to pocket nine colored billiard balls in ascending numerical order (1-ball, 2-ball, … 9-ball). An individual frame is won by the player pocketing the last ball on the table which is the 9-ball. The player to win a predetermined number of frames first wins the game. Each frame of 9-ball games begins with a break shot. The purpose of the break shot is to separate the racked object balls and to pocket at least one ball so that the player can remain on the table. If the player misses, the visit (which consists of a series of consecutive successful shots) will be passed to the opponent.

It is generally believed that a powerful break shot can separate the object balls well such that the player can pocket the subsequent object balls easily and continue staying on the table until he/she wins by pocketing the 9-ball. Therefore, the break shot represents an important shot in 9-ball, and a good break shot can logically increase the chance of winning the game. Jeanette Lee, a professional 9-ball player ranking number 1 in 1990s, once shared that “*the break is the most important shot in nine-ball … because it can give you control of the table*” (Lee and Gershenson, 2007) (p. 102). It is further stated that “*the best breaks in nine-ball spread the rack*” (p. 102). Regarding how a player executes the break, it is advised that “*when you break, try to get the cue ball to hit the 1-ball as solidly as possible, so it will roll off about a foot and stop (stops near the center of the table)*” (p. 103). Learning from her personal experience, a “good” break shot leads to the cue ball stopping near the center of the pool table and the object balls widely spread. In general, a player is expected to win the frame after a good break shot in professional tournaments. Furthermore, clearing the table is a specialized case of winning a frame where the player who takes the break shot pots all colored balls in sequence to win the frame without letting the opponent play at all. Players take advantages of a good break shot in some tournaments where the frame winner continues to break in the following frame (Shepard, 1997). In the Turning Stone Classic, for example, a player who always clears the table after a good break shot tends to keep winning without passing the play to the opponent. Despite some anecdotal evidence and experts’ opinions, there is no scientific literature on how the break shot may impact game outcomes in cue sports.

Currently, there is no established method to quantify the characteristics of a break shot. Based on coaching expert’s opinion, the position of the cue ball and the distribution of the remaining colored balls after the break are of key interest. To evaluate the ball positions and movements on the pool table, previous studies on cue sports (Haar and Faisal, 2020; Haar et al., 2020; Pan et al., 2021) applied 2D video analysis which involved a digital camera and analysis software. Based on video analysis, one could quantify the end position of the cue ball after the break to examine if parking the cue ball near the center of the pool table would indeed indicate a good break shot as suggested by the anecdotal evidence. Regarding how well the object balls are spread by the break shot, no previous studies have attempted to analyze the ball distribution pattern on the pool table after the break. One possible approach is to adopt the Voronoi diagrams which is a partitioning of a 2-D plane based on the “nearest-neighbor” rules (Aurenhammer, 1991) assigning each dot to a corresponding cell. As a computational geometry method, Voronoi diagrams have been widely applied in various disciplines (Fonseca et al., 2012; Lopes et al., 2017; Sun et al., 2018; Xiao et al., 2018). In civics and planning, Xiao et al. (2018) used Voronoi diagrams to describe the pedestrian motion patterns wherein each pedestrian was represented by a point in a Voronoi cell. In informatics, Voronoi diagrams were applied to partition the target region for further study of wireless local area network (Sun et al., 2018). In biology, Voronoi diagram was proposed as a new method to characterize the geometrical distribution of human cells (Lopes et al., 2017). In team sports, such as Futsal, Voronoi diagrams have been utilized to identify players’ distribution patterns where each player on the pitch was treated as a dot with the coordinates (x, y) (Fonseca et al., 2012). In 9-ball, because the total dimension (the playing surface) is finite, an optimal spread of the balls on the table should lead to similar Voronoi cell areas. This would mean that a small standard deviation of the Voronoi cell areas among all the object balls would indicate that balls are spread evenly. Thus, Voronoi diagrams may be suitable tools for analyzing ball distribution pattern in cue sports by generating cells for the remaining balls on the table after the break.

This study aimed to quantify the characteristics of the break shot from two aspects: (1) the end position of the cue ball, and (2) the distribution of the balls remaining on the pool table after the break shot. Secondly, as the break shot is considered important in 9-ball, the relationship between the characteristics of the break shot and frame outcomes was also examined. Based on the professional player’s experience, it was hypothesized that a good break shot would be characterized by the cue ball positioned close to the center of the table and the remaining balls widely spread across the table. A good break shot was also hypothesized to increase the chance of potting more balls immediately after, clearing the table, and winning a frame in professional 9-ball tournaments.

## Method

### Data

This study involved analyses of publicly available online videos and was approved by the Nanyang Technological University Institutional Review Board (Protocol Number: IRB-2019-05-42). Videos of the semifinals and finals of World Pool Billiard Association (WPA) ranked tournaments in the years of 2019 and 2020^1^ were downloaded from the Internet. The finals and semifinals of six tournaments including 2019 Diamond Las Vegas Open (Men), 2019 International 9-ball Open (Men), 2019 World 9-Ball Championship (Men and Women), 2019 World 9-Ball Championship China Open (Men and Women), 2019 WPA Players Championship (Men), and 2020 Turning Stone Classic XXXIII (Men) were analyzed in this study. There were 275 frames in total (241 from men’s tournaments and 34 from women’s tournaments) after removing the frames where the cue ball fell into the pocket or jumped out of the pool table, because the relative positions of the cue ball could not be measured in those situations. Player rankings were within the top 50 for male players and top 5 for females according to the official rankings provided by WPA in January 2021 (see text footnote 1).

### Data Processing

**FIGURE 1:**
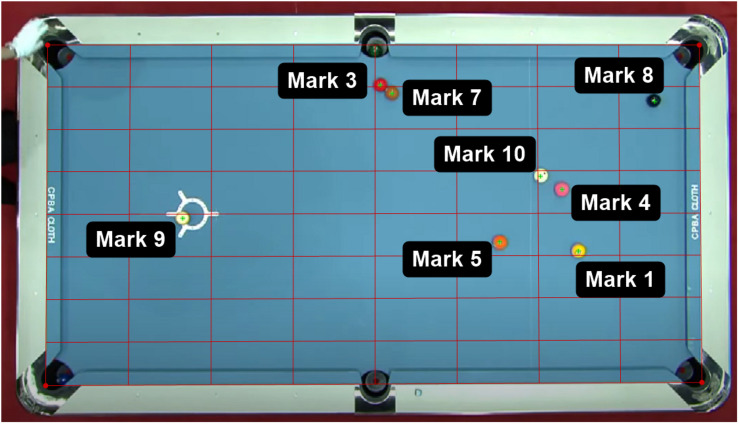
Example of 2D video analysis in Kinovea, where a “perspective grid” (red) was applied to calibrate the pool table and the “mark” (green) was added in the center of each ball. Marks 1–9 indicates 1-ball to 9-ball; Mark 10 indicates the cue ball; 2-ball and 6-ball went into the pocket and therefore not shown. Video was extracted from publicly available source (https://www.youtube.com/watch?v=2sEqz5N0cug).

Top-view videos of professional 9-ball tournaments were downloaded and analyzed using Kinovea (version 0.8.27, available for download at: http://www.kinovea.org), a 2D motion analysis software which has shown good accuracy in measuring objects at an angle range of 45° to 90° (Puig-Diví et al., 2019). In a previous study on 9-ball test protocols, excellent inter- and intra-rater reliability was found in measuring ball movements from video recordings using Kinovea (Pan et al., 2021). After each break shot, the positions of all balls remaining on the pool table were digitized manually (Figure 1). Firstly, the “perspective grid” was applied to calibrate the pool table and was set as 127 cm × 254 cm (Pan et al., 2021) which was the dimension of a standardized pool table used in professional tournaments. Then, the “mark” tool was implemented to mark each of the ball remained on the table. The 2D coordinates (x, y) of all balls remaining on the table were obtained for analysis.

The first variable for the characteristics of the break shot is the cue ball distance (CBD, in cm), defined as distance between the end position of the cue ball and the center of the pool table. The CBD was calculated using the equation (1),

(1) $$CBD=\sqrt{{\left(x_{10}-x_{0}\right)}^{2}+{\left(y_{10}-y_{0}\right)}^{2}}$$

where x_10_ and y_10_ were the x and y coordinates of the cue ball (Mark 10), respective; x_0_ and y_0_ were the x and y coordinates of the center of the pool table. To examine the inter-rater and intra-rater reliability, a sub-sample of 20 out of the 275 frames were randomly chosen. To examine the inter-rater and intra-rater reliabilities, a sub-sample of 20 out of the 275 frames were randomly chosen. This sub-sample was independently digitized by two members of our research team, and repeated digitized twice by one team member, to obtain the CBD values.

**FIGURE 2:**
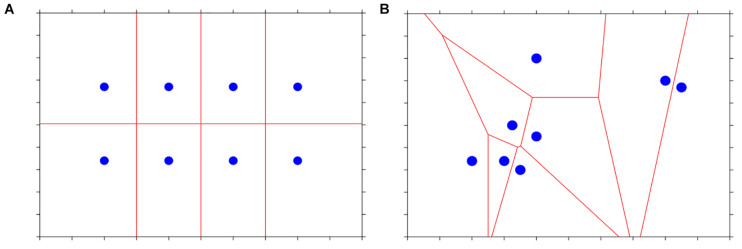
Schematic representations of Voronoi diagram. **(A)** Dots (blue) are evenly distributed leading to a small standard deviation of cell areas. **(B)** Co-existence of both big and small cells causing a big standard deviation of cell areas.

Voronoi diagram was applied to analyze the ball distribution after the break shot using the MATLAB function (v2020b, MathWorks, Natick, MA, United States) proposed by Ong (2011). In a Voronoi diagram, each of the remaining balls on the table (blue dot in Figure 2) was assigned to a cell (polygon, shaped by the red edges and table boundaries in Figure 2). Once the area of each cell was obtained, the standard deviation of the areas of all cells (SDVD, in cm^2^) was computed. A smaller SDVD (similar areas among the Voronoi cells, Figure 2A) would imply that the balls on the table were more evenly distributed as the areas occupied by each ball were similar. Likewise, a larger SDVD (different areas among the Voronoi cells, Figure 2B) would indicate that some balls were clustered, and others were far from the clusters.

During the break shot, the number of balls falling into pockets was counted. The player needs to pocket at least one ball during the break in order to stay on the table for the next visit. The following game outcomes were also obtained for each frame: (1) if the player who took the break shot cleared the table or not, (2) if the player won the frame or not, (3) the number of balls the player potted in the next visit immediately after the break shot. Table clearance is a specialized case of winning a frame where the player makes ball(s) pocketed during the break shot and then pots all object balls legally in one consecutive visit without give the opponent any chance to play. Theoretically, a desirable break shot should allow the player to pot more balls in the next visit and may even clear the table and win the frame.

### Statistical Analyses

Data are expressed as mean (standard deviation). Inter-rater and intra-rater reliabilities were examined using intraclass correlation coefficient (ICC) on SPSS (version 26.0, IBM Corp., Armonk, United States). ICC was interpreted as *slight* (<0.20), *fair* (0.21–0.40), *moderate* (0.41–60), *substantial* (0.61–0.80), or *almost perfect* reliability (>0.80) (Altman, 1991; Heng et al., 2016). Standard error of measurement (SEM) was calculated from the ICC results using the formula: SEM = SD × √(1−ICC). All other statistical analyses were performed on JASP (version 0.14.1; JASP Team, 2020) statistical software. The association between the number of balls potted into pockets in the break shot and SDVD was assessed using Spearman’s rho (r_*s*_) since the assumption of normality was violated. Spearman’s rho (r_*s*_) was also performed to test the associations between the characteristics of the break shot (CBD and SDVD) and the frame outcomes (i.e., the number of balls potted in the subsequent shots). Binary logistic regression was run to identify the significant characteristic indicators, with two dependent variables of match outcomes set as Clear = 1 and Not = 0, and Win = 1 and Not = 0. Odds ratios (OR) and corresponding 95% confidence intervals (95% CI) were presented (Robertson and Joyce, 2018). Statistical significance was set at the 0.05 level.

## Results

**FIGURE 3:**
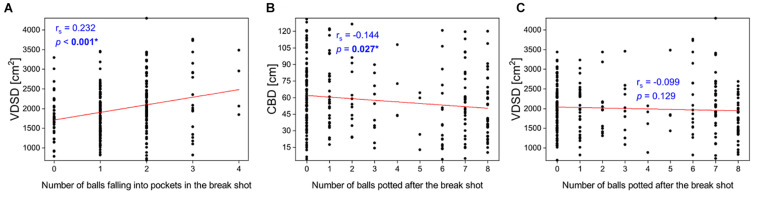
**(A)** Correlations between the number of balls falling into pockets in the break shot and SDVD. Correlations between the number of balls potted after the break shot and **(B)** CBD and **(C)** SDVD. CBD denotes the distance between the end position of the cue ball and the center of the pool table; SDVD denotes the standard deviation of the areas of all the Voronoi diagram cells. Significant difference (*p* < 0.05) is shown in bold text and indicated by an asterisk.

Results of all 275 digitized videos showed an average of 58.9 (30.7) cm for CBD, and 1975.6 (641.8) cm^2^ for SDVD. In the break shot, 1.4 (0.8) balls were potted into the pockets. After the break shot, 3.1 (3.3) balls were potted in the subsequent visit. The results of ICC indicated *almost perfect* inter-rater (ICC_2, 2_ = 0.972, SEM = 4.4 cm) and intra-rater (ICC_2, 1_ = 0.999, SEM = 0.8 cm) reliabilities. Spearman correlation analysis indicated a significant positive association between the number of balls falling into pockets after the break shot and SDVD (*r*_*s*_ = 0.232, *p* < 0.001, Figure 3A). Also, a significant negative association between CBD and the number of balls potted in the subsequent visit was identified (*r*_*s*_ = −0.144, *p* = 0.027, Figure 3B). Conversely, no significant association between SDVD and the number of balls potted after the break shot was found (*p* = 0.129, Figure 3C).

Regarding the frame outcome, Clear the table or Not, the binary logistic regression model was statistically significant [χ^2^(2) = 6.616, *p* = 0.037]. The model as a whole explained between 2.4% (Cox & Snell *R*^2^) and 3.4% (Nagelkerke *R*^2^) of the variance in the frame outcome for “Clear or Not,” and correctly classified 70.5% of frames (0% for “Clear” and 100% for “Not”). Of the independent variables that were included in the model, only CBD made a unique statistically significant contribution to the model (*p* = 0.012, Table 1). Binary logistic regression identified no significant predictor of the game outcomes for “Win or Not” [χ^2^(2) = 1.722, *p* = 0.423].

**TABLE 1:** Binary analysis of the two predictors of the break shot characteristics for the game outcomes in 9-ball.

	Clear or not			Win or not		
Predictors	χ^2^	OR (95% CI)	*p*	χ^2^	OR (95% CI)	*p*
CBD	6.268	0.989 (0.980, 0.998)	0.012*	0.119	0.999 (0.991, 1.007)	0.730
SDVD	0.032	1.000 (1.000, 1.000)	0.859	1.588	1.000 (1.000, 1.001)	0.208

*CBD denotes the distance between the end position of the cue ball and the center of the pool table; SDVD denotes the standard deviation of the areas of all the Voronoi diagram cells. χ^2^ denotes Wald’s chi-square, OR denotes odds ratio, and CI denotes confidence interval. Significant difference (p < 0.05) is shown in bold text and indicated by an asterisk.*

## Discussion

This study aimed to quantify the characteristics of the break shot in 9-ball and its relationship with the outcomes of a frame. The novel contributions of the current study were that (1) we pioneered the application of Voronoi diagrams in cue sports to objectively quantify the characteristics of the break shot, and (2) we established the relationship between the break shot characteristics and the frame outcomes. Building on professional 9-ball player’s experience (Lee and Gershenson, 2007), the cue ball position (indicated by CBD) and ball distribution on the table (indicated by SDVD) were proposed to represent important break shot characteristics. This study investigated only professional tournament and elite players to purposely avoid the situations where less-skilled players performed a good break shot but were unable to clear the table due to their relatively lower playing levels.

### Balls Potted During the Break Shot

Having balls pocketed in the break shot allows the player to remain on the table and continue to play. Logically, it is desirable to have more balls falling into pockets during the break since having fewer balls remaining on the table will give the player a higher chance to clear the table and win the frame. Results of this present study showed a significant positive correlation between the number of balls potted during the break and SDVD. This indicates that the more balls falling into the pockets during the break, the more clustered the remaining object balls on the table. Although the association is weak (*r*_*s*_ = 0.232), having clustered balls on the table may not be a good situation for the next shots. If the player cannot clear the table due to the clustered balls blocking each other, the visit will be passed to the opponent. This finding suggests that it may not be a good strategy for players to pocket many balls in the break shot *per se* because they may face difficulties to continue pocketing the remaining balls that are clustered close to each other. Players should aim for potting at least one ball during the break to stay on the table and be aware that potting more balls is not necessarily better for subsequent shots.

### Cue Ball Position

After the break shot, the current study found a significant correlation between CBD and the number of balls potted immediately after the break. This result reaffirmed the significance of the end position of the cue ball, revealing that when the cue ball was parked near the table center the player could pot more balls within the same visit. This finding is not surprising and confirms the anecdotal guidelines proposed by players and coaches. When planning for a break shot, players are advised to park the cue ball near the center of the pool table after the break to set up for the next shot.

Similar to studies on other sports in which important factors contributing to successful game outcomes were identified (Gómez et al., 2013; Rumpf et al., 2017; Robertson and Joyce, 2018), the current study examined the relationship between the break shot characteristics and the frame outcomes using binary logistic regression. For instance, in Australian Rules football, opposition rank and match location were two significant predictors found to be associated with the match outcomes (Win or Loss) (Robertson and Joyce, 2018). When investigating the matches in the 2014 FIFA World Cup Brazil, it was reported that shot accuracy was the best predictor for match success (Rumpf et al., 2017). In elite basketball, different predictors were identified for men and women for predicting ball possessions (Gómez et al., 2013). In the present study on cue sport, it was found that CBD was a significant predictor for the game outcome, “Clear or Not.” Although the prediction accuracy was 70.5% overall, the model correctly predicted 100% of cases where the table was not cleared, but 0% of cases of table clearance (i.e., all object balls were pocketed within one visit following the breaks shot). This suggests that CBD is not successful in predicting table clearance following the break shot. Similarly, CBD also failed to predict the game results “Win or Not.”

The complexity of 9-ball may explain why cue ball position alone could not predict table clearance or winning of a frame. It should the noted that the success of 9-ball games depends on many factors, such as the players’ skills, strategies, experience, and even luck. An excellent break shot alone may not significantly contribute to the success of the remaining visits and outcome of the frame. For example, a player may have skillfully parked the cue ball at the table center in the break shot but the next object ball (lowest numbered ball on the table) is blocked by other balls. Failing to pot the next object ball would force the player to pass the play to the opponent and may then lose the frame eventually. In addition, players may choose to deliver a “safety shot” under situations when they are not confident in winning or making table clearance. A “safety shot” does not aim to pot the any object ball in the pocket but to place the cue ball in a challenging position for the opponent. The frequent use of “safety shot” in the profession tournaments was evident, as reflected by the high occurrence of “zero” in Figure 3 which shows that no balls were potted after the break shot in 46.5% of the time (128 out of 275 frames). Thus, while CBD could provide the information about the relative position of the cue ball after the break shot, this variable alone is insufficient to indicate the likelihood of success in 9-ball games. More variables, such as the object balls distribution may be needed to comprehensively quantify the break shot characteristics.

### Ball Distribution

Voronoi diagram as a computational geometry method (Aurenhammer, 1991) measures the surrounding areas of the balls left on the table after the break shot such that it shows if there are clusters of balls by comparing the areas among the cells. Similar to previous studies (Lopes et al., 2017; Sun et al., 2018; Xiao et al., 2018), the current study treated each ball as a simple dot regardless of its number when applying the Voronoi diagram to calculate the cell areas. It was hypothesized that a small SD of the areas (i.e., indicating that the balls are evenly distributed), which is a desirable game situation, would increase the chance of clearing the table and winning the frame. However, this hypothesis was not supported by the results of the present study. Ball distribution (indicated by SDVD) was not related to the number of balls potted after the break, nor did it predict whether the player could clear the table or win the frame. It is acknowledged that while Voronoi diagram describes the ball distribution, the relative positions of the cue ball and the next object ball to be potted are not taken into account. It is possible that the next ball to be potted is blocked by other balls despite the balls were sufficiently separated and showing a small value of SDVD. Future studies should therefore refine the current analysis to examine the balls distribution and at the same time considering also the relative positions of the cue ball to the next ball to be potted on the table (i.e., for the subsequent shot).

Although this study showed that Voronoi diagrams may be too simplistic for the application in 9-ball break shots, the method of quantifying object distribution may be useful in other situations. When applied to team sports, for example, it would be interesting to examine whether a more “spread” or “compact” players formation may lead to better performance. It is also possible to investigate if factors, such as fatigue and playing level of the opponents would influence the position distribution of team sports players.

### Limitations

There are a few limitations to this study that can be identified. Firstly, we only considered the number of balls potted in the visit immediately following the break. This approach does not reflect the game dynamics comprehensively because a frame may involve many visits played alternatively between the two players before one of them wins. Future work can extend the analysis to all visits played in a frame to better understand how a break shot can impact the playing characteristics of the entire frame. Secondly, the sample investigated in the current study was delimited to professional tournaments and elite players and therefore the established association between the break shot characteristics and the frame outcomes may not be directly applied to less-experienced players. Thirdly, the sample size of 275 frames may not have fully captured the diverse playing styles and break shot characteristics among professional players. Hence, an investigation into a large number of frames in 9-ball is warrant. Lastly, the present study included much fewer women’s games (34 frames) compared with men’s games (241 frames) and therefore all frames were treated in the same group. As the game characteristics and playing strategies may differ between sexes, future studies could compare the break shot and game outcomes between male and female players.

## Conclusion

In professional 9-ball tournaments, pocketing more balls during the break is associated with more clustered balls remaining on the table. Parking the cue ball near the table center after the break can be a good strategy to facilitate pocketing more balls immediately after. Table clearance and winning of a frame are likely influenced by multiple factors and could not be predicted by the break shot characteristics alone. While Voronoi cell areas could provide an objective measure of the ball distribution on the table, this method did not reveal any association between ball distribution and game outcomes. Future work should consider more in-depth analysis of the object balls distribution after the break shot, taking into account the relative positions of the cue ball and the next object ball to be potted on the table.

## Data Availability Statement

The original contributions presented in the study are included in the article/Supplementary Material, further inquiries can be directed to the corresponding author/s.

## Ethics Statement

The studies involving human participants were reviewed and approved by the Nanyang Technological University Institutional Review Board. Written informed consent for participation was not required for this study in accordance with the national legislation and the institutional requirements.

## Author Contributions

PK, JK, and JP originated this project and performed statistical analysis. SS and JP processed and analyzed the data. All authors discussed the results and actively contributed to the final manuscript.

## Conflict of Interest

The authors declare that the research was conducted in the absence of any commercial or financial relationships that could be construed as a potential conflict of interest.

## Publisher’s Note

All claims expressed in this article are solely those of the authors and do not necessarily represent those of their affiliated organizations, or those of the publisher, the editors and the reviewers. Any product that may be evaluated in this article, or claim that may be made by its manufacturer, is not guaranteed or endorsed by the publisher.

## Footnotes

1
https://wpapool.com/ranking/
